# Leaf Classification for Crop Pests and Diseases in the Compressed Domain

**DOI:** 10.3390/s23010048

**Published:** 2022-12-21

**Authors:** Jing Hua, Tuan Zhu, Jizhong Liu

**Affiliations:** 1 School of Software, Jiangxi Agricultural University, Nanchang 330045, China; 2 School of Mechatronics Engineering, Nanchang University, Nanchang 330031, China

**Keywords:** agricultural images, image classification, neural networks, compressed domain

## Abstract

Crop pests and diseases have been the main cause of reduced food production and have seriously affected food security. Therefore, it is very urgent and important to solve the pest problem efficiently and accurately. While traditional neural networks require complete processing of data when processing data, by compressed sensing, only one part of the data needs to be processed, which greatly reduces the amount of data processed by the network. In this paper, a combination of compressed perception and neural networks is used to classify and identify pest images in the compressed domain. A network model for compressed sampling and classification, CSBNet, is proposed to enable compression in neural networks instead of the sensing matrix in conventional compressed sensing (CS). Unlike traditional compressed perception, no reduction is performed to reconstruct the image, but recognition is performed directly in the compressed region, while an attention mechanism is added to enhance feature strength. The experiments in this paper were conducted on different datasets with various sampling rates separately, and our model was substantially less accurate than the other models in terms of trainable parameters, reaching a maximum accuracy of 96.32%, which is higher than the 93.01%, 83.58%, and 87.75% of the other models at a sampling rate of 0.7.

## 1. Introduction

Agricultural pests and diseases have always been one of the major impediments to crop yields and, each year, pest and disease problems lead to significant crop yield reductions. According to the Food and Agriculture Organization of the United Nations, up to 40 percent of global crop yields are lost to pests each year, and invasive insects cause at least USD 70 billion in damage. Plant pests that damage economically important crops are becoming more destructive and increasingly pose a threat to food security and the environment as a result of climate change.

Wheat, corn, and rice are the three major food crops in the world. Among them, corn ranks third in the world in terms of planted area and production, after wheat and rice, and second in China after rice. Widely grown in tropical and temperate regions around the world, maize has a high nutritional value and is an excellent food crop and one of the indispensable raw materials for food, health care, light industry, and chemical industry [1]. Maize can suffer from a variety of diseases during growth that can seriously affect its value and cause economic losses to growers. For example, common ones include corn caudex leaf spot (GLS), corn stripe blight (NLB), and corn rust (Rust).

Therefore, addressing pest and disease problems is a top priority during crop growth. In the process of managing pests and diseases, how to identify pests and diseases is the first step of treatment. However, professional knowledge of pests and diseases is held by experts in the field of agriculture, and the vast majority of crop growers do not have the ability to identify and distinguish between pests and diseases. Moreover, often agricultural experts are unable to arrive on site in a timely manner, while limited manpower cannot carry out pest and disease identification over large areas, and there is a degree of manual misjudgment. Therefore, some methods to help growers distinguish between pests and diseases are able to help growers distinguish between pests and diseases in the first place and achieve the purpose of control.

Among them, the use of neural networks for image identification of pest and disease leaves has become hot research in the industry. With the development of deep neural networks, many models using CNN for classification have emerged. J Ma [2] et al. used deep CNN for feature recognition of four classes of cucumber leaf diseases. Kawasaki [3] et al. introduced a recognition system for cucumber leaves, which was also implemented based on CNN. G.L. Grinblat [4] et al. used a three- to six-layer CNN to classify three legumes. Ahmad [5] et al. used four different convolutional neural networks for comparative trials, where Iception V3 had the best performance. Bi [6] et al. showed that ResNet152, Inception V3, etc., were used to identify apple leaf spot and rust models collected by agricultural experts, where ResNet152 had the highest accuracy of 77.65%. Jiang [7] et al. first segmented four rice diseases using Mean Shift algorithm, then extracted shape features by manual computation, extracted color features using CNN, and, finally, identified the diseases using SVM classifier, and the results showed that the accuracy of CNN using segmentation algorithm was 92.75% and 82.26% for those that were not applicable. Long [8] et al. used the AlexNet model for training for classifying Camellia sinensis diseases, using two schemes, migration learning and training from scratch, respectively, and the results showed that migration learning has better classification performance and faster convergence speed.

Therefore, to solve the above problem, image recognition of various types of pests and diseases is needed to identify the correct pests and diseases to solve the problem. However, conventional neural networks perform a full sampling when processing data, i.e., classify the original size of the image. If a compressed perception algorithm that can compress the data size is used for classification, the classification task is roughly divided into three steps in compressed perception:

- The signal object to be classified is first observed and sampled;
- Reconstruction of the sampled data by reduction;
- Classification of the reconstructed data.

However, in step 2, the reconstruction of the signal often takes up a lot of time and resources [9]; what if the reconstruction is skipped directly? That is, directly classify the compressed acquired data directly; then, this will greatly reduce the use of time and resources. However, the signal also needs to satisfy the property of sparsity in the acquisition process, but not all signals are sparse [10]. Moreover, the observation matrix and the sparse representation basis have to be incoherent in nature. Therefore, the design of the sampling matrix and the properties of the signal also have certain requirements in the compressed perceptual classification task [11].

With the rapid development of deep learning (DL), more and more neural networks have been applied to various fields, and a number of deep learning models related to compressive perception have been proposed. In 2015, Mousavi et al. first combined deep learning with compressed perception and proposed a stacked denoising autoencoder (SDA) based on self-encoder for capturing the statistical correlation between signals in different elements to improve the quality of reconstructed signals [12]. Kuldeep Kulkarni et al. first combined compressive sensing with CNN and proposed ReconNet, a noniterative chunked compressive sensing reconstruction network based on convolutional neural networks, which aims to implement a noniterative and fast compressive sensing reconstruction algorithm, but the network itself does not contain a sampling module [13]. Lyu Meng et al., in 2017, combined deep learning and ghost imaging reconstruction algorithms to build a deep neural network model, which led to a further improvement in the quality of the reconstruction [14]. Moreover, in 2017, Mousavi et al. proposed Deeplnverse network, aiming to solve the problems of incomplete sparsity of data under a fixed change base and slow convergence of traditional algorithms with high reconstruction accuracy [15]. Yao et al. proposed Dr2-Net for further enhancement of Reconnet by combining ReconNet with the idea of residuals of ResNet [16]. Shi W et al. proposed CSNet, which uses a deep residual convolutional neural network to sample and reconstruct images, building a three-stage network structure with compressed sampling, initial reconstruction, and deep reconstruction [17]. To achieve efficient storage and fast encoding of the sampling matrix, M. Iliadis et al. combined the binary neural network (BNN) in the field of model compression with compressed sensing and proposed the DeepBinary-Mask model [18].

CSNet as a depth model applied to image compression perception seeks a new path for the traditional CS approach. In the neural network, all the data are processed by normalization with uniformity. Some of the original signals are no longer required to be sparse; they can be one-dimensional signals with temporal correlation or two-dimensional image signals [19]. In neural networks, we use convolutional and fully connected layers instead of sampling matrix, so that there are learnable parameters under each sensing rate (SR), which can be learned automatically in the neural network. Therefore, in this paper, we propose a novel compression module applied to neural networks for image classification in the compressed domain using neural networks as a tool and compression-aware algorithms as a basis; our contributions are listed below:

- We propose a block consisting of three two-dimensional convolutional layers and an up-sampling layer to replace the part of the sampling matrix in the traditional CS, which we call CS-Block, and we place it at the head of the network model, and the number of output features can be adaptively varied according to the sampling rate (adaptive change according to the input sensing rate).
- Based on the above CS-Block module, a model CSB-Net for compression domain classification is proposed to better fit in the compression domain. The network has a smaller number of parameters, reducing the required time for training and saving the use of resources. After the features are extracted in the convolutional layer, the extracted features are reinforced using the channel attention mechanism. We selected the most suitable structure for the current network from SE-Net [20], ECA [21], and CBAM [22], and used full connection layer [23] and drop-out [24] layers for the classification phase, modifying the number of input and output channels to better fit the compression domain classification task.
- We have conducted experiments in different crop pest leaves with better applicability. We finally compared four network models and can see that our method has the best classification performance, having the highest accuracy among these models.

In this paper, the second section is Preliminary and contains some of the methods and models used. Section 3 is Materials and Methods, which contains the dataset collection and our proposed method. Section 4 is Results, which contains the setup parameters and the results of our experiments. Section 5 is Discussion, which contains our discussion of the results in the paper and derives some relevant results. Section 6 is Conclusion, which contains our summary of the paper and some prospects for future work.

## 2. Preliminary

### 2.1. Compressed Sensing

Compressive sensing (CS) theory was proposed by DDonoho, Emmanuel Candes, and Tao, a Chinese scientist, in 2007 [25,26,27,28,29]. In conventional compressive sensing data acquisition, the original signal is acquired by multiplying the signal by a designed measurement matrix and, later, the original signal is obtained from the measured signal by solving the optimization problem. The signal is required to satisfy the RIP property at the time of acquisition [30] and Equation (1) is the constrained isotropy condition, i.e., for any constant there are:

(1) $$\left(1-\delta_{k}\right){\left\|C\right\|}_{2}^{2}\leq {\left\|\emptyset^{C}\right\|}_{2}^{2}\leq \left(1+\delta_{k}\right){\left\|C\right\|}_{2}^{2}$$

Under this theory, the sampling rate of the signal no longer depends on the bandwidth of the signal but on the structure and content of the information in the signal, so that it satisfies the properties of (1) whether the signal is sparse or not, and (2) the sampling system is uncorrelated with the observation system. The process of compression can be expressed as Equation (2):

(2) y=ϕx

where y is the measured signal, ϕ is the measurement matrix of M × N (M << N), and *x* is the original signal. The measured signal y can be reconstructed when only ϕ satisfies Equation (1) and when x has the property of sparsity in some domains.

(3) x=ψα

The original signal *x*, when it has the sparse property, can be expressed as a set of orthogonal bases Ψ= [$\psi_{1},\psi_{2},\cdot \cdot \cdot ,\psi_{n}$], where α is the expression of the signal *x* under the orthogonal basis Ψ. If α has k (k << N) non-zero elements, we consider α as k-sparse under the orthogonal basis Ψ, and the measured signal can be reconstructed only when *x* is sparse or α is sparse.

### 2.2. AlexNet

**Figure 1:**
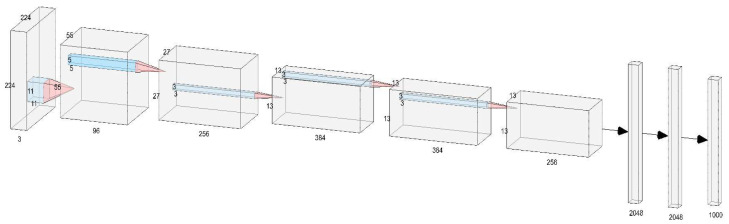
AlexNet model structure diagram.

In 2012, AlexNet was launched [31]. Alex Net uses an eight-layer convolutional neural grid and won the ImageNet 2012 image recognition challenge by a large margin. The AlexNet model is a landmark model that is the dividing line between shallow and deep neural networks. The network model in this paper refers to the AlexNet model to some extent, and its structure is shown in Figure 1.

### 2.3. Attentional Mechanisms

#### 2.3.1. SE-Net

**Figure 2:**
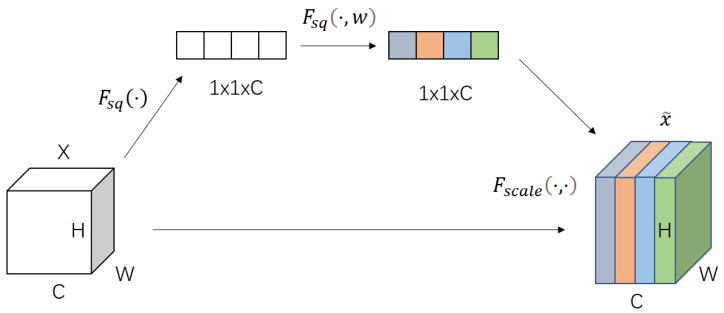
Before inputting the SE attention mechanism (left colorless figure C), the importance of each channel of the feature map is the same. After passing SE-Net (right color figure C), different colors represent different weights, making the importance of each feature channel become different, making the neural network focus on certain channels with large weight values.

The Squeeze-and-Excitation Networks (SE-Net) attention mechanism is a mechanism for adding attention to the channel dimension, and the key operations are divided into squeeze and excitation. The model uses the importance level to assign a weight value to each feature, thus allowing the neural network to pay more attention to certain feature channels. Squeeze part (colorless plot of 1 × 1 × C in Figure 2) compresses each 2D feature (H*W) into one real number by averaging pooling layers, converting the feature map haste [h,w,c] into [1,1,c], while the excitation part (colorful plot of 1 × 1 × C in Figure 2) produces a weight to each feature channel, constructs the inter-channel correlation by two fully connected layers’ correlation, and the number of output weights is the same as the number of channels in the input feature map. In the scale part, the normalized weights obtained earlier are weighted to the features of each channel. The structure of the SE-Net model is shown in Figure 2.

#### 2.3.2. Efficient Channel Attention (ECA)

**Figure 3:**
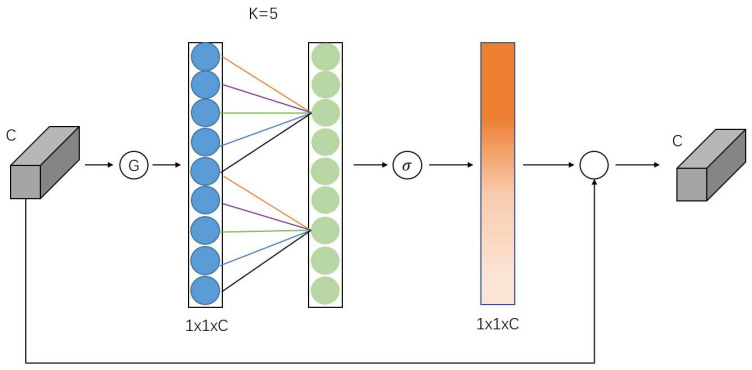
ECA attention mechanism structure diagram.

ECANet is an implementation of the channel attention mechanism, and ECANet can be seen as an improved version of SE-Net. The proponents showed that dimensionality reduction in SE-Net can have a negative effect on the channel attention mechanism and that capturing the dependencies between all channels is inefficient and unnecessary. In the ECA module, the input feature map is globally averaged and pooled, and the feature map C is transformed from a matrix of [h,w,c] to a vector of [1,1,c], and the adaptive one-dimensional convolution kernel size k is calculated based on the number of channels of the feature map, after which k is used in the one-dimensional convolution to obtain the weight for each channel of the feature map, and the normalized weights are multiplied with the original input feature map channel by channel to generate the weighted feature map. The normalized weights and the original input feature map are multiplied channel by channel to generate the weighted feature map C. The ECANet structure is shown in Figure 3.

#### 2.3.3. CBAM

CBAM attention mechanism is composed of channel attention mechanism and spatial attention mechanism. CBAM introduces two analysis dimensions of spatial attention and channel attention mechanism from two scopes, channel and spatial.

**Figure 4:**
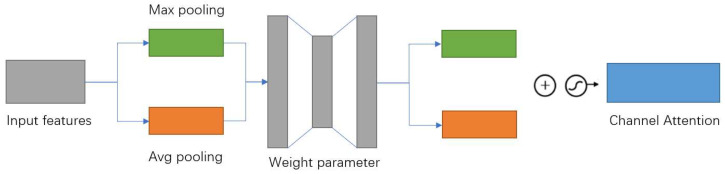
Channel attention mechanism in CBAM.

In the channel attention mechanism, the input feature maps are first subjected to global maximum pooling and global average pooling, respectively, to obtain two feature descriptions with different dimensions. After that, the number of channels is first dropped by a fully connected layer, and then recovered by another fully connected one. Finally, the weights of each channel of the sigmoid activation function feature map are normalized, and the normalized weights are multiplied with the input feature map to obtain the final realized channel attention (blue square in Figure 4).

**Figure 5:**
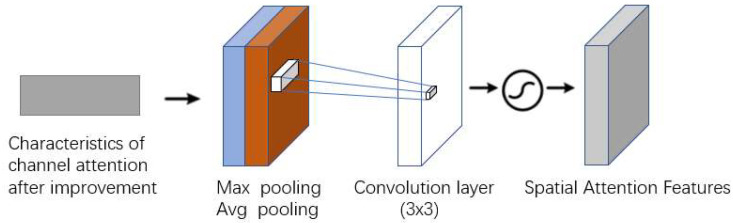
Spatial attention mechanism in CBAM.

In Figure 5 spatial attention mechanism module, the input feature map is first conducted with maximum pooling and average pooling in the channel dimension, and the two feature maps after pooling are stacked in the channel dimension. Then, the feature map shape is changed from [b,2,h,w] to [b,1,h,w] by fusing the channel information using a 3 × 3 convolution kernel. Finally, the convolved result is normalized to the spatial weights of the feature map by the sigmoid function, and then the input feature map and the weights are multiplied together.

Where spatial attention allows the network model to focus more on the regions of the image that play a role in the classification results, channel attention is used to deal with the assignment relationships of the feature map channels.

## 3. Materials and Methods

### 3.1. Dataset

**Figure 6:**
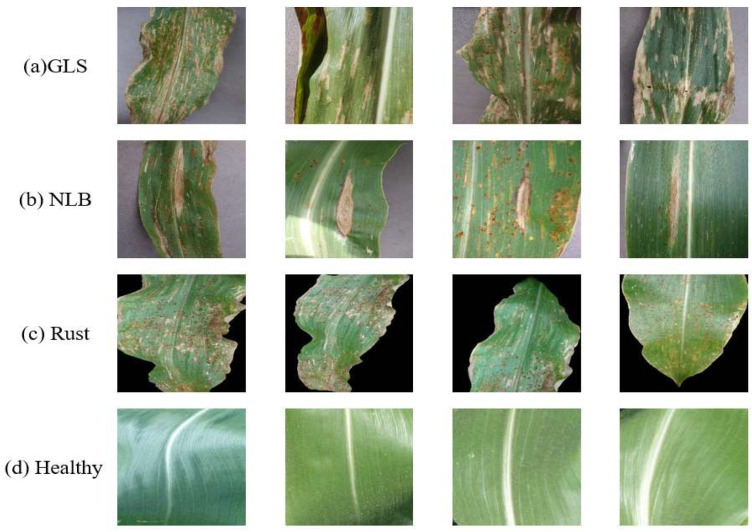
Images of the four types of maize leaves classified in this paper: (**a**) gray leaf spot of maize (GLS), (**b**) corn leaf spot (NLB), (**c**) corn rust (Rust), and (**d**) healthy maize leaves.

The dataset in this paper was intercepted from the Plant Village dataset [32], the Plant Village dataset contains 54,303 images of healthy and diseased, divided into 38 classes, and contains data of a large number of common crops. In this paper, four different corn leaf classifications were used from the Plant Village dataset, including three classes of corn pest and disease leaves and one class of healthy corn leaves, with a total of 4142 images, of which 3313 were used as the training set and 829 as the validation set. As shown in Figure 6 for the unprocessed images, the 3 categories of pests and diseases are (a) gray leaf spot of maize (GLS), (b) corn leaf spot (NLB), and (c) corn rust (Rust), and 1 category is (d) healthy corn leaves. The number of each class of maize pest and disease images is shown in Table 1.

### 3.2. Image Preprocessing and Labeling

**Figure 7:**
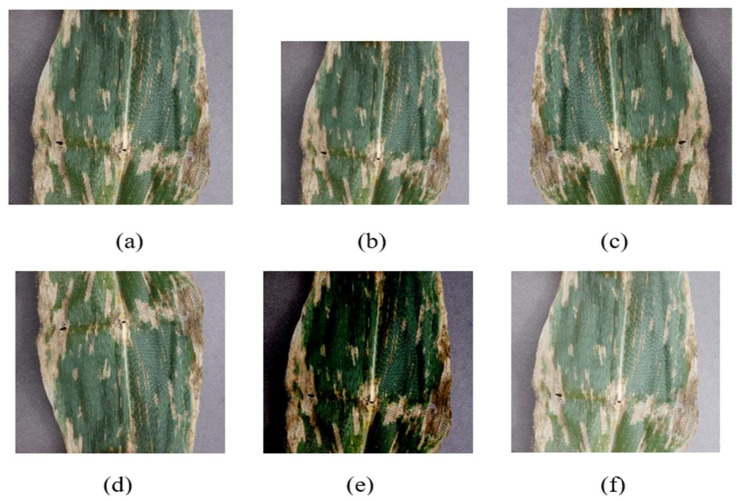
Image data after preprocessing: (**a**) original image; (**b**) reconstructed as 224px × 224px image; (**c**) horizontally flipped image; (**d**) vertically inverted image; (**e**) image with 30% brightness reduction; (**f**) image with 30% brightness enhancement.

The dataset contains diseased maize leaves, as well as healthy maize leaves, and is normalized to a 256 px × 256 px RGB image. In order to improve the generalization ability of the network for classification [33], we will preprocess the collected dataset. First, it is reconstructed as a 224 px × 224 px × 3 RGB image, random horizontal flipped with 50% probability, random vertical flipped with 50% probability, the brightness of the image reduced, and the brightness of the image enhanced. Using processed images for training improves the robustness of the model. As shown in Figure 7, the pre-processed image data are shown.

### 3.3. Proposed Methodology and Model

**Figure 8:**
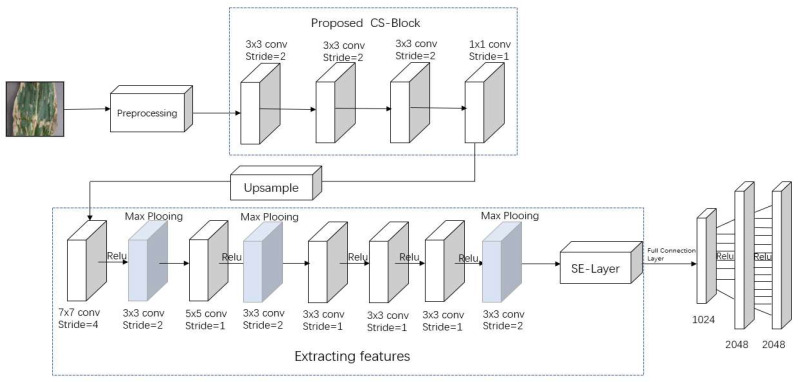
The overall framework.

In this paper, we propose a neural network model CSBNet for crop pest and disease leaf classification. The model is divided into four parts: compression part, extracted features part, enhanced extracted features, and image classification. The original image, when it enters the network, needs to go through a preprocessing session first. After first performing a uniformized reconstruction of the pixel size, and then horizontal flip, after the normalization process, its value range is made to be between [0, 1]; this will speed up the convergence of the network, improving the robustness of data, and, also, give a better response to the processing of activation functions. Then, CS-Block is entered for compressed sampling, preventing under-sampling by up-sampling layers again, making the sampled data smaller than the size of the convolution kernel, and the resulting inability to perform subsequent convolution operations [34]. The corresponding features are extracted by entering the feature extraction layer, and then adding features to the feature map through the SE-Net layer; finally, the classification is conducted through the fully connected layer. The following subsections provide a detailed description of the model structure section. Figure 8 shows our proposed model.

### 3.4. Preprocessing

At the time the original image is input to the model, the normalization operation needs to be completed. Before training, to avoid excessive order of magnitude differences in the variables of each input, which influence the effect of subsequent algorithms, the data will generally be normalized to a decimal number between [0, 1] first [35]. The mathematical expression of the normalization is as in Equation (4):

(4) $$x_{norm}=2\;*\;\frac{x-x_{min}}{x_{max}-x_{min}}-1$$

where x is the original data and $x_{norm}$ is the normalized data. After normalization, the value of the input data is between [0, 1] and contributes to the convergence of the model, avoiding the polarization of weights, thus causing numerical problems.

### 3.5. Proposed CS-Block

In the traditional CS method, the measurement matrix is divided into a random observation matrix and a deterministic observation matrix, and is subject to the RIP property. We used 4 sequential convolutional layers and 1 up-sampling layer instead of the measurement matrix in the traditional CS method. Because the convolution is representable as a matrix-to-matrix multiplication [36], a sequential 4-layer convolution formula is (5):

(5) $$y=\sum (\omega_{4}\sum \left(\omega_{3}\sum \left(\omega_{2}\sum \left(\omega_{1}x_{norm}+b_{1}\right)+b_{2}\right)+b_{3}\right)+b_{4}\;\;\;\;\;\;\;\;\;\;\;\;\;=\omega_{4}\omega_{3}\omega_{2}\omega_{1}x+\omega_{4}\omega_{3}\omega_{2}b_{1}+\omega_{3}\omega_{2}b_{2}+\omega_{3}b_{3}+b_{4}$$

where $\omega_{i}$ and $b_{i}$ are the weight and bias in the ith convolutional layer in the CS-Block, respectively. The convolution can be expressed as a linear representation of the original signal x, so convolution can replace the traditional CS sampling matrix in the network model to sample and compress the information.

In Figure 8, we can see that the first three convolutional layers have a kernel size of 3 × 3 of convolution and stride is 2, the last layer of convolution has a kernel size of 1 × 1 and stride is 1, and the number of output filters is X, then X = 192 × Sensing Rate (Sensing Rate is the ratio of the sampled data to the original data). Because the processed data are an RGB image, the input filter is 3, the output filters for the first three layers are all 64, and compression only is guaranteed, without changing the dimensionality of the data. Before entering the extracted feature layer, it is necessary to go through the up-sample layer to prevent the sampled data from being too small due to convolution; data are smaller than the size of the convolution kernel, resulting in the inability to perform feature extraction.

### 3.6. Feature Extraction

We build on top of the AlexNet model; first, the value of the input filter for the first layer of convolution is changed and the quantity value of the CS-Block output, that is, X (X = 192 × Sensing Rate). In addition, the convolution kernel of 7 × 7 is chosen in the first layer, stride is 4, and the number of filters is 48. Because the ReLU function is more expressive for linear functions, in particular, it is reflected in the deep network, and ReLU is constant due to the gradient of the non-negative interval, as in Equation (6). So, it can solve the gradient disappearance problem of sigmoid function and the convergence of the model is maintained in a steady state. So, the measured values are mapped linearly to the output using the Relu activation function.

(6) $$f\left(x\right)=max\left(0,\;x\right)$$

Then, the max pooling layer is used for feature extraction, the pooling kernel is 3, and stride is 2. The features of the image after 5 layers of convolution and 3 layers of pooling, after the image is converted to SE-Net structure for enhanced feature extraction, give more weight to some task-related channels and increase to improve the accuracy of classification.

### 3.7. Classification

**Figure 9:**
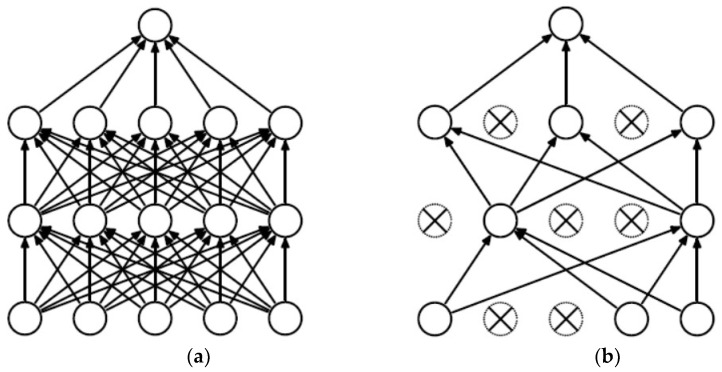
(**a**) Standard neural net, (**b**) after applying dropout.

In this model, we classify by building fully connected layers and dropout layers. Fully connected layers mainly map the learned “distributed feature representation” to the sample labeling space and provide weighting of the extracted features. In the proposed model, we use three fully connected layers for classification, with input dimensions of 512, 1024, and 2048; the final feature channels with weights are output by the function. Each fully connected layer is preceded by a dropout layer to randomly disconnect 50% of the neurons; this is used to improve the generalization ability of the model, enhance the robustness of the model classification, and prevent overfitting. The working schematic is shown in Figure 9.

## 4. Results

In this subsection, we compare and contrast the attention mechanisms under fixed SR. Comparing the classification performance of network models under different SR, each of these models incorporates our proposed CS-Block. The contrasting network models are: (1) CS-AlexNet model with CS-Block added, (2) our proposed network model, (3) ResNet50 network model with CS-Block added [37], and (4) ResNet50-SE. After comparison, it is found that our proposed model obtains the best performance in terms of evaluation metrics.

### 4.1. Experimental Parameter Setting

The hardware environment for this experiment was conducted on Windows 10 OS with Intel(R) Core(TM) i7-11800H 2.3 Ghz CPU, 16 G RAM, and an RTX 3060 to accelerate the training. For the software environment, the compiler used was PyCharm Community Edition, the Python version used was 3.10, the Pytorch version was 1.11.0, and the CUDA version was 11.3.

#### 4.1.1. Training Parameters Setting

Our proposed method and comparative trials were validated on the Plant Village dataset. We selected four leaf states of maize, three of them are diseased and one kind healthy. There are 4142 images in total, of which 3313 are the training set and 829 are the validation set. In our experiments, the sensing rate (SR) is defined as:

(7) $$SR=\frac{x}{y}$$

where *x* is the data of the original image sampled by CS-Block and *y* is the original image data. In the present experiment, our selected sensing rates in this experiment are 0.05, 0.1, 0.2, 0.3, 0.4, 0.5, 0.6, and 0.7.

In order to calculate the difference between the forward computation of each iteration of the neural network and the real result, which will guide the correct direction for the next step of training, we introduce the cross-entropy loss function; the loss function measures the degree of difference between two different probability distributions in the same random variable, and the smaller the value of cross-entropy, the better the prediction of the model is. Equation (8) represents the distance between the actual output (probability) and the desired output (probability), that is, the smaller the value of cross-entropy, the closer the two probability distributions are. Suppose the probability distribution p is the desired output, the probability distribution q is the actual output, and $\left(x\right)$ is the probability distribution of either p or q. This loss function is given by the following equation:

(8) $$H_{\left(p,q\right)=}-\sum_{x}\left(p\left(x\right)\right)log\left(q\left(x\right)\right)$$

Optimizers are used to update and calculate network parameters that affect model training and model output, making the approximation reach the optimal value. In this paper, the Adam optimizer is selected and the learning rate (LR) set to 0.0001. Adam Optimizer combines the advantages of both AdaGrad and RMSProp algorithms. It is computationally efficient and has low memory requirements.

#### 4.1.2. Evaluation Indicators

After setting the training parameters for the network, the data are put into the net model for training and each neuron is updated with the weights of the neurons by whether the output of the forward propagation algorithm is equivalent to the label. After repeating the current operation, however, we do not know whether the model has learned new capabilities and whether the performance of the model has improved. Therefore, we introduce accuracy, precision, recall, and F1 score performance metrics. First of all, the confusion matrix is described by introducing the elements of correct or incorrect classification prediction, which consists of four elements: true positive (TP, predict positive samples to positive samples), false positive (FP, predict negative samples to positive samples), true negative (TN, predict negative samples to negative samples), and false negative (FN, predict positive samples to negative samples). They are calculated by the following formula:

(9) $$\mathrm{Accuracy}=\frac{\mathrm{TP}+\mathrm{TN}}{\mathrm{TP}+\mathrm{FP}+\mathrm{TN}+\mathrm{FN}}$$

In the model prediction, the proportion of elements belonging to the positive sample outcome are predicted as:

(10) $$\mathrm{Precision}=\frac{\mathrm{TP}}{\mathrm{TP}+\mathrm{FP}}$$

In the prediction model, the proportion of elements belonging to the positive sample outcome that are correctly predicted is:

(11) $$\mathrm{Recall}=\frac{\mathrm{TP}}{\mathrm{TP}+\mathrm{FN}}$$

The value of F1 is precision (P) and recall ®:

(12) $$F1=\frac{2}{\frac{1}{P}+\frac{1}{R}}=\frac{2\times P\times R}{P+R}$$

Among the above evaluation indicators, the larger the value of accuracy, precision, and recall, the better. When within a certain range, when the value of F1 is small, the better the generalization performance of the training model. When training and evaluation are completed, the training model has better performance.

### 4.2. A Comparison of Different Attention Mechanisms

In this paper, we chose SE-Net, ECA, and CBAM for our model performance enhancement, and we used the proposed model and ResNet50 to add CS-Block on the premise of adding SE-Net, ECA, and CBAM for comparison experiments, respectively. Moreover, to control the variables to ensure the accuracy of the experiments, we choose a fixed SR to determine the final experimental results.

The comparison test classification accuracy results of our proposed model adding SE-Net, ECA, and CBAM are shown in Table 2. We selected the sensing rate at SR = 0.7 for the test, the dataset was selected from four classifications of maize pest and disease leaves for the experiment, the number of correctly predicted categories is shown in the table, and the total number of samples validated was 816. In Table 2, “Classification accuracy of our proposed model when adding SE-Net, ECA, and CBAM”, the number of correctly detailed categories in each category is also shown. We can see our proposed model achieves a total accuracy of 96.32%. The accuracy reached 95.22% when the model of enhanced feature extraction was replaced with ECA; when CBAM is used, the accuracy reaches 95.47%.

**Figure 10:**
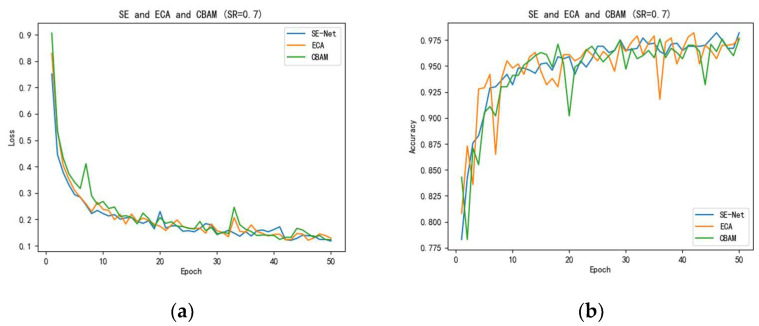
(**a**) Epoch and loss, (**b**) epoch and accuracy.

From Figure 10a, we can see that, when SE-Net is added, the curve of loss will be smoother and converge relatively fast, while ECA and CBAM are more oscillating and, finally, SE-Net has a lower loss. In Figure 10b, SE-Net has a higher accuracy curve.

Table 3 shows the comparison of attention mechanisms between the ResNet50 models with the addition of CS-Block. When ResNet50 was added to SE-Net, the accuracy reached 87.75%. When ResNet50 was added to ECA, the accuracy reached 84.44% and, when ResNet50 was added to CBAM, the accuracy reached 86.27%. When SE-Net is added, it is higher than the other two attention mechanisms by 3.31% and 1.48%, respectively; therefore, SE-Net has the highest accuracy for the current classification task, which is why we choose the SE-Net module to enhance the feature extraction. Through the above two sets of comparison experiments, we can see that SE-Net has a strong capability in strengthening features by compressing two-dimensional features into one real number through the built-in global average pooling to generate the weight values connecting two feature channels and, finally, the weights are automatically learned by the fully connected network according to the loss function, which makes the weights of effective feature channels larger to achieve better classification. The result is better classification.

Figure 10a shows the training and loss relationship plots for the above three attention mechanisms when SR is taken as 0.7, and Figure 10b shows the training and accuracy relationship plots for the above three attention mechanisms when SR is taken as 0.7.

### 4.3. Comparison of Parameters of Different Network Models

The experiments in this section show the comparison of the number of parameters between the models. The reduction in the number of parameters results in less training time and a smaller resource footprint. Moreover, the model with low number of parameters is closer to the usage scenario of traditional compressed perception. In addition, the CS-Block proposed in this paper and the added SE-Net attention mechanism improve the accuracy by 3% of percentage with less space occupation. Table 4 shows the parameter sizes of the AlexNet model, the proposed model (No SE-Net), our proposed model, the ResNet50 model with CS-Block added, and the ResNet50-SE model with CS-Block added, respectively.

Table 4 shows the comparison of the number of parameters for each type of model. From Table 4, we see that our proposed model is only 48.66 MB, which only increases the total size of 0.01MB compared to the proposed model (No SE-Net), and, after the experiments, in the later subsections, we prove that it improves the classification accuracy and performance by 3% or even more when SR takes the value of 0.7, and the overall performance is higher. The deep networks with more layers produce larger training data than other deep learning models because of the deepening layers of these networks, making the size of ResNet50 up to 407.39 MB, and then, after adding SE-Net, it reaches a size of 459.52 MB, while the total number of parameters of our proposed model with a size of 48.66 M is much smaller than the other compared models.

As a result, we can conclude from the experimental results that the CSBNet complex model has a smaller number of parameters, adding only 0.1 MB size of parameters after adding the SE-Net module, while the overall performance of the model is improved. Having fewer training parameters can speed up the training time and save the total time of the whole classification task, as well as save hardware resources, making it useful on more mobile platforms. Fewer resources can be used on more micro platforms, broadening the application area.

### 4.4. Performance Comparison of Different Network Models

We compare our proposed model with the CS-AlexNet model, and the ResNet50 model with CS-Block added and the ResNet50-SE model with CS-Block added for comparison. All four models were performed on a four-classified maize pest leaf dataset, of which there are 104 for GLS, NLB has 197 sheets, Rust has 238 sheets, Healthy has 290 sheets, and the total number of images verified is 829. Moreover, comparing the accuracy results of the eight SRs we obtained and some performance, these eight SR values are: 0.05, 0.1, 0.2, 0.3, 0.4, 0.5, 0.6, and 0.7.

**Figure 11:**
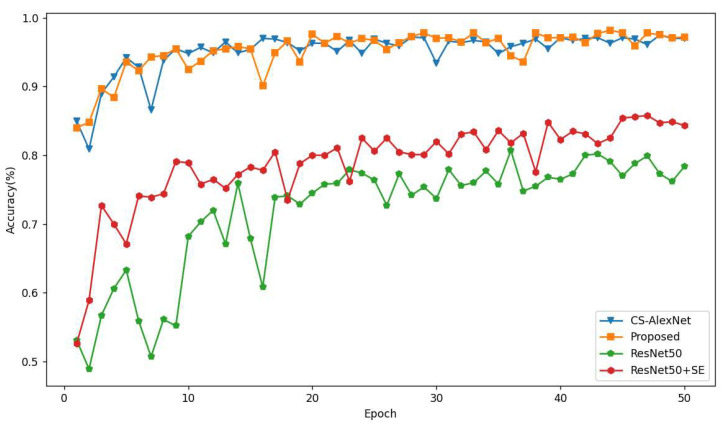
Accuracy trend of 4 model validation sets when SR takes the value of 0.7.

Figure 11 shows the trend graph of the accuracy of the validation set when SR is 0.7. During the training process, as the model is trained more often, the accuracy slowly plateaus and our proposed model (orange) reaches a maximum of 97.2%, which is higher than the 96.3% of CS-Block, 78.4% of ResNet50, and 84.3% of ResNet50 + SE, with better classification performance.

According to Table 5 and Table 6, it can be concluded that our proposed model has better classification accuracy; an accuracy of 96.32% was achieved. Our proposed model is much more accurate than the ResNet50 model with 83.58% and 87.75%. SE-Net, in the face of SR taking small values, when processing dimensional features of low sampling rate images, has side effects of the giving channel attention mechanism because of its own downscaling mechanism and, instead, did not properly capture the connection between channels, resulting in weight shifts that do not favor the positive aspects of the classification task, resulting in reduced classification accuracy. In addition, when SR takes a high value, when processing dimensional features of high sampling rate images, more information about the features obtained enables SE-Net to more accurately capture the connection between channels and performance of enhanced features, thus improving the classification accuracy.

**Figure 12:**
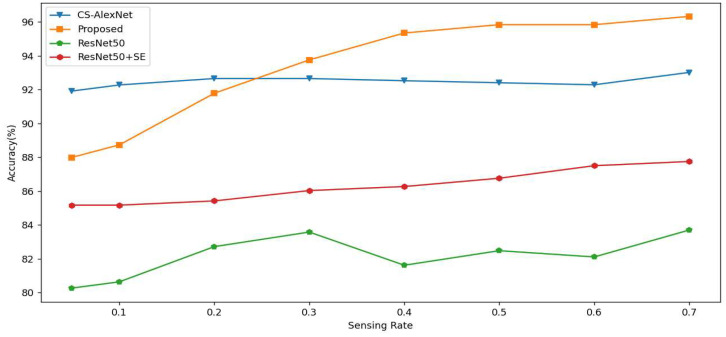
Model accuracy trends in the test set with SR = [0.05, 0.1, 0.2, 0.3 … 0.7].

In Figure 12, when SR is taken as 0.7, our proposed model achieves 96.32% accuracy, which is higher than the 93.01% accuracy of the CS-AlexNet model without the attention channel, while ResNet50 and ResNet50-SE only achieve 80.27% and 87.75% accuracy, which is lower than the accuracy of our proposed model. The result curve of classification at the full sampling rate shows a positive trend with the sampling rate, and SR has a positive correlation with the accuracy of classification. This is in accordance with the logical relationship in traditional CS, i.e., when the SR value is taken as large, the better the reconstructed object is, and, when the SR value is taken as small, the effect is the opposite.

Table 6 shows the performance summary of each model for all sampling rates, and the performance results in the table are taken as the average of the results for each sampling rate. It can be more intuitively seen that the proposed model has an overall higher performance than the other models, and also has a better classification performance for the whole dataset.

### 4.5. Experimental Results for Another Dataset

**Figure 13:**
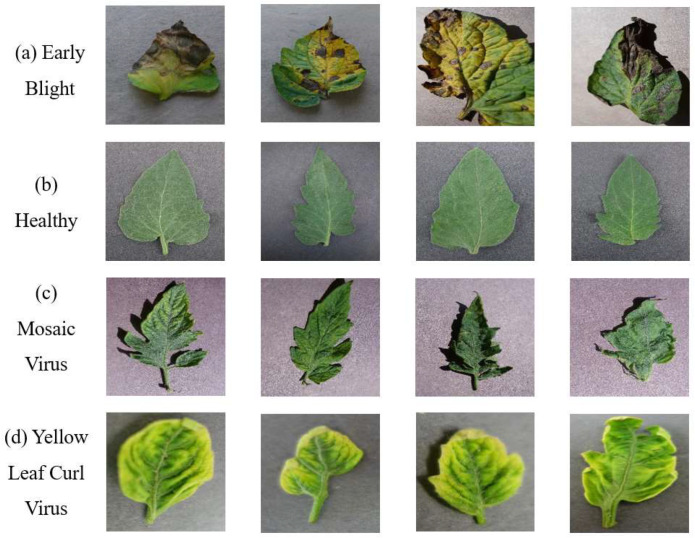
Tomato pest and disease leaves.

The model can be trained on different datasets to verify the robustness of the model. Therefore, we selected another dataset of pest and disease leaves, another dataset was selected on Plant Village for tomato pest and disease leaves, and four leaf categories are included: tomato early blight, tomato mosaic virus, tomato yellow leaf curl, and tomato healthy leaf. In this dataset, a training set of 1000 sheets for each category, a total of 4000 sheets are composed. The validation set consists of 200 sheets per class, composed of 800 sheets in total; this allows for a uniform distribution of training samples. The tomato dataset is shown in Figure 13.

**Figure 14:**
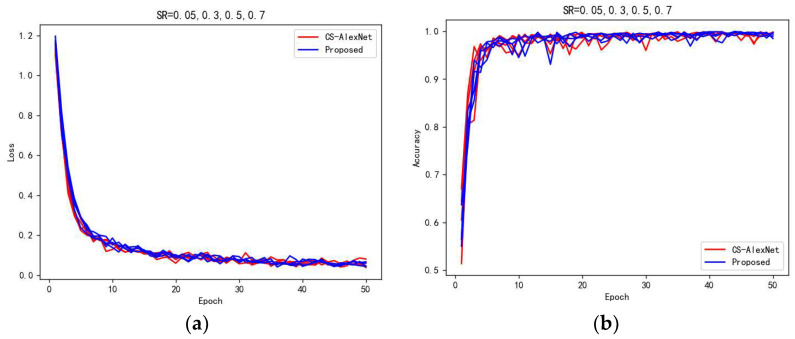
(**a**,**b**) shows the trend of the relationship between training epoch, loss, accuracy, etc.

In the experiment, in a comparison using CS-AlexNet model and our proposed model, the values of SR are: 0.05, 0.1, 0.2, 0.3, 0.4, 0.5, 0.6, and 0.7, respectively. Figure 14 shows the comparison of epoch and loss values, and epoch and accuracy values when the SR values are 0.05, 0.3, 0.5, and 0.7, respectively. In Table 7, the highest accuracy of our proposed model reaches 88.13% and the average accuracy reached 82.13%; this is higher than the 81.73% accuracy of CS-AlexNet.

This experiment demonstrates the robustness of our proposed model by comparing two different databases, both of which have the best accuracy of our proposed method.

### 4.6. Comparison of Different Learning Rates

The learning rate exists as a very important parameter in the training process of neural network models. The size of the learning rate affects the rapid convergence of the mode and when to converge to the minimum value. A suitable learning rate value allows the model to converge to a local minimum in a suitable amount of time; it is beneficial for our model training. Therefore, we selected three different values of the learning rate to conduct a comparison test in order to derive a more suitable learning rate value for our proposed model.

**Figure 15:**
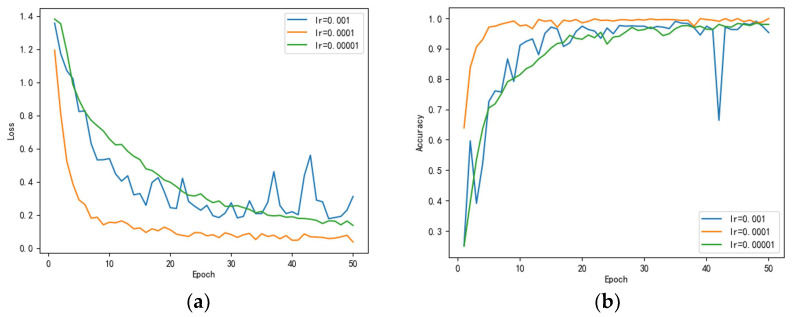
(**a**) Loss of each learning rate (**b**) accuracy of each learning rate.

We set the learning rates to 0.001, 0.0001, and 0.00001, respectively, for comparison. Figure 15 shows the results of our learning rate comparison experiments under the proposed model; we can see in Figure 14a that, when the learning rate is equal to 0.0001, the speed and degree of model convergence is significantly higher than the learning rate of 0.001 and 0.00001. In addition, as can be seen by Figure 14b, the accuracy at a learning rate of 0.0001 is significantly higher than that at 0.001 and 0.00001. Therefore, in our proposed model, we chose to set the learning rate to 0.0001 to train the model, which accelerates the convergence of the model more rapidly and enhances the learning ability of the model at the same time.

## 5. Discussion

From the experimental results, we can see that our proposed CS-Block compression model is robust and highlights good performance in the comparison among different network models. Robustness experiments were also conducted on different datasets to demonstrate the adaptability of the proposed model. Traditional compression-aware algorithms, on the other hand, often rely on optimization algorithms to improve better performance, and a single optimization algorithm has a limited impact on its own results. The model with the combination of compressed sensing and neural networks, on the other hand, adds more trainable parameters, giving it more parameters to improve and iteratively optimize the model, allowing us to get closer to the results we want and, therefore, achieve better performance.

Our proposed method accomplishes the whole classification work by compressing the data, extracting features, reinforcing features and classifying them. Combined with the various experimental results in this paper, the proposed model CSBNet, which has fewer trainable parameters than other models while training on compressed collected data, is able to improve the processing efficiency of the network by processing fewer original data than traditional neural networks. Fewer training parameters and smaller models are also beneficial to run on a wider variety of hardware platforms, broadening the application scenarios of plant pest and disease leaf classification.

## 6. Conclusions

In this paper, we propose a network structure model with compressed sampling for identifying data objects of pest and disease leaves. Compared with traditional neural networks, our model reduces the number of trainable parameters for the whole model, will take less resources during training, processes data faster, and innovatively can recognize images in a compressed domain with good performance. A channel attention mechanism SE-Net is added between the feature extraction and fully connected layers, which improves the dimensionality of the fully connected layer compared to the AlexNet model and increases the accuracy of recognition, making it more applicable to compressed domain image classification. Experiments are also conducted on two different datasets to ensure the robustness of the model. In the Plant Village dataset, the classification results achieved up to 96.32% accuracy for corn pest leaves and up to 88.13% accuracy for tomato pest leaves. After our experiments, we proved that our proposed model has better classification recognition ability and can have better adaptability on different pest and disease leaves, and shows better robustness for the recognition of different pest and disease leaves. However, we found that the classification accuracy of the model is low when the SR is taken to be low. In future work, we will focus our attention on improving the classification effect at low SR taking values.

## Figures and Tables

**Table 1:** Number of test leaf pest and disease images.

Class	Number of Training Images	Number of Testing Images
GLS	409	104
NLB	788	197
Rust	954	238
Healthy	1162	290
Total	3313	829

**Table 2:** Classification accuracy results of the proposed model adding SE, ECA, and CBAM (SR = 0.7).

Class	Proposed (SE)	Proposed + ECA	Proposed + CBAM
GLS	103	100	95
NLB	163	157	164
Rust	232	232	232
Healthy	288	288	288
Total	786	777	779
Accuracy	96.32%	95.22%	95.47%

**Table 3:** Classification accuracy results of the proposed CS-Block with SE, ECA, and CBAM in ResNet50, respectively (SR = 0.7).

Class	ResNet50 + SE-Net	ResNet50 + ECA	ResNet50 + CBAM
GLS	38	34	45
NLB	166	172	164
Rust	231	216	215
Healthy	281	267	280
Total	716	689	704
Accuracy	87.75%	84.44%	86.27%

**Table 4:** Comparison of the proposed model with other models in terms of number of parameters.

Parameters Type	Proposed	AlexNet	Proposed(No SE-Net)	ResNet50	ResNet50-SE
Total params	6,003,328	62,378,344	6,001,144	25,846,056	28,377,048
Trainable params	6,003,328	62,378,344	6,001,144	25,846,056	28,377,048
Forward/backward pass size (MB)	25.19	11.09	25.18	308.22	350.69
Params size (MB)	22.90	237.95	22.89	98.59	108.25
Estimated Total Size (MB)	48.66	249.62	48.65	407.39	459.52

**Table 5:** Comparison of the accuracy of the proposed model with other models at different values of SR.

SR	Accuracy (%)			
	CS-AlexNet	ResNet50	ResNet50-SE	Proposed
0.05	91.91	80.27	85.17	87.99
0.1	92.27	80.64	85.17	88.73
0.2	92.65	82.72	85.42	91.78
0.3	92.65	83.58	86.03	93.75
0.4	92.52	81.62	86.27	95.34
0.5	92.4	82.48	86.76	95.38
0.6	92.28	82.11	87.5	95.38
0.7	93.01	83.70	87.75	96.32

**Table 6:** Performance of the proposed model compared to other models.

	CS-AlexNet	ResNet50	ResNet50-SE	Proposed
Average Recall (%)	90.29	72.62	77.47	90.78
Average F1-Score (%)	91.36	77.08	81.62	91.97
Average Accuracy (%)	92.46	81.71	86.26	93.08
Maximum Accuracy (%)	93.01	83.58	87.75	96.32

**Table 7:** Identification accuracy of tomato pest and disease leaves.

Models	SR								Average Accuracy (%)
	0.05	0.1	0.2	0.3	0.4	0.5	0.6	0.7	
CS-AlexNet	69.13	78.25	81.13	82.13	82.25	82.88	85.25	86.38	80.92
Proposed	74.25	80.63	80.25	80.88	82.25	84.63	86.00	88.13	82.13

## Data Availability

Publicly available datasets were analyzed in this study. These data can be found here: (https://plantvillage.psu.edu/, accessed on 12 April 2016). The source code implementing the proposed method in this paper is available in the url: (https://github.com/zt0528/CS-Block-model, accessed on 19 November 2022).
